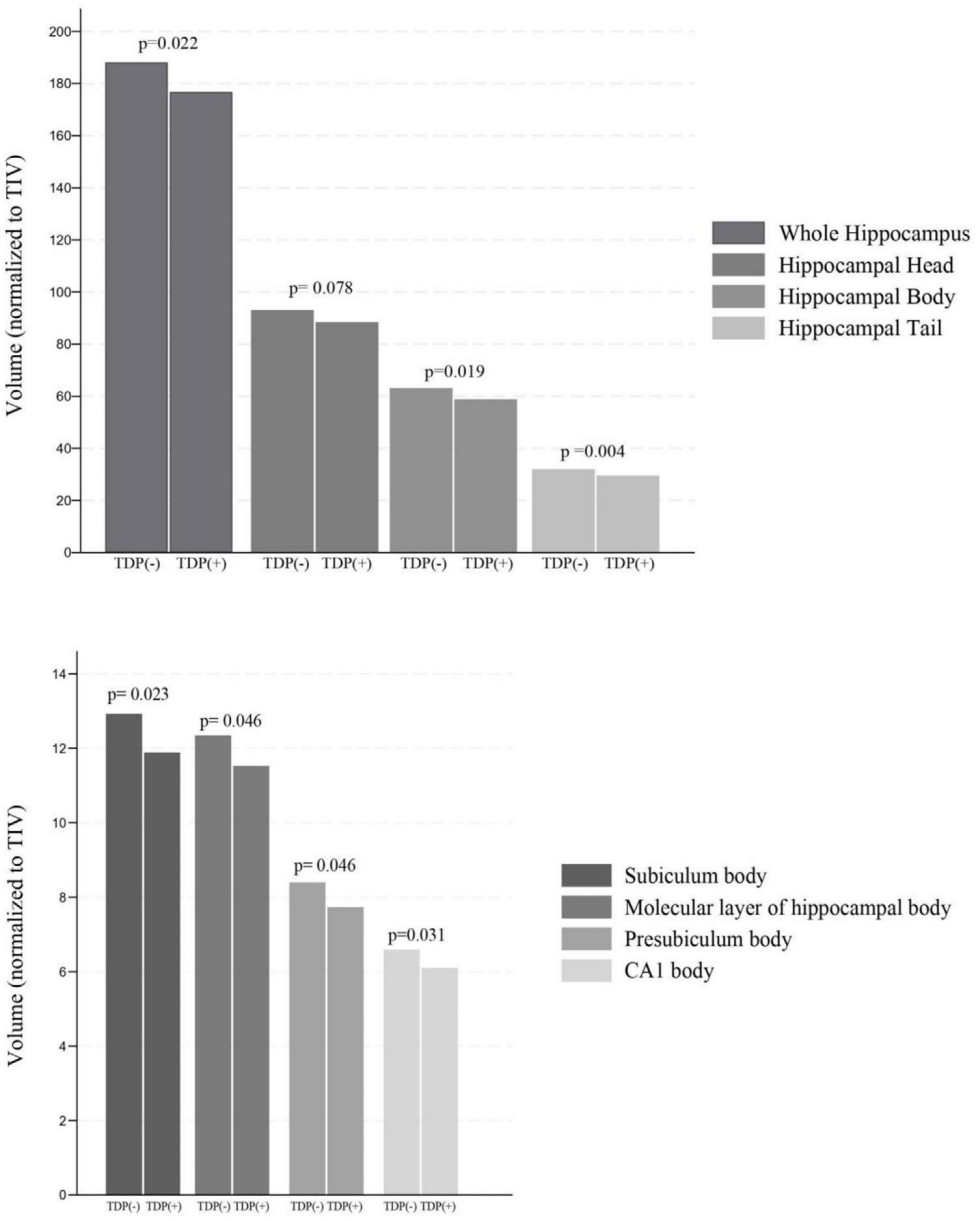# TDP‐43 association with Subiculum and CA1 Hippocampal Subfield Atrophy in Primary Age‐Related Tauopathy

**DOI:** 10.1002/alz.093812

**Published:** 2025-01-09

**Authors:** Hossam Youssef, Rodolfo G. Gatto, Nha Trang Thu Pham, Ronald C. Petersen, Mary M. Machulda, Ross R. Reichard, Dennis W. Dickson, Clifford R. Jack, Jennifer L. Whitwell, Keith A. Josephs

**Affiliations:** ^1^ Mayo Clinic, Rochester, MN USA; ^2^ Mayo Clinic, ROCHESTER, MN USA; ^3^ Department of Laboratory Medicine and Pathology, Mayo Clinic, Rochester, MN USA; ^4^ Department of Neuroscience, Mayo Clinic, Jacksonville, FL USA; ^5^ Department of Radiology, Mayo Clinic, Rochester, MN USA

## Abstract

**Background:**

The TAR DNA‐binding protein of 43 kDa (TDP‐43) is linked to hippocampal volume loss and faster rates of hippocampal atrophy in Alzheimer's disease and primary age‐related tauopathy (PART). Hence, TDP‐43 is becoming an important player in age‐related neurodegeneration. To advance our understanding of TDP‐43 effect on hippocampus, we conducted an imaging‐pathological study to determine which specific hippocampal subfields are affected by TDP‐43 in cases of PART.

**Method:**

One‐hundred fifteen autopsied cases from Mayo Clinic Alzheimer Disease Center, Study of Aging and Neurodegenerative Research Group were analyzed. All had completed antemortem brain MRIs, underwent neuropathological examination per NIA and Alzheimer’s Association (NIA‐AA) criteria and had additional assessment for TDP‐43 deposition. Only cases meeting criteria for PART (Thal phases 0‐2 and Braak NFT stages I‐IV) were included. Hippocampal subfield segmentation was performed using FreeSurfer‐7. Brain volumes were normalized to total intracranial volume (TIV) when comparing TDP‐43 groups. Statistical analyses including logistic regression analyses were utilized to explore associations between TDP‐43 status and hippocampal subfield volumes.

**Result:**

Forty‐nine of the 115 cases (43%) with PART were female. Thirty‐seven (32%) cases were TDP‐positive. Mean age at last MRI was 84 years. There were no differences in pathological characteristics between both groups except for presence of hippocampal sclerosis which was more frequent in the TDP(+) group (P <0.001). Whole hippocampal volume was significantly smaller in the TDP(+) group (176 ±31 vs. 188 ±27, p=0.049). After stratification of hippocampus into head, body, and tail, TDP‐positive cases had smaller volumes of hippocampal body (p=0.031) and tail (p=0.021) but did not reach significance for hippocampal head (p=0.129). When regions were subdivided into hippocampal subfields, TDP(+) cases showed smaller volumes of molecular layer of the hippocampal body (p=0.046), CA1 body (p=0.031), subiculum body (p=0.023), and presubiculum body (p=0.046) compared to TDP(‐) cases. Logistic regression analysis adjusting for TIV confirmed these associations.

**Conclusion:**

This study underscores the association between TDP‐43 and the hippocampus and provides further evidence that TDP‐43 targets multiple regions and subfields of the hippocampus in patients with PART. Intriguingly, regions targeted are those where TDP‐43 deposition is microscopically observed, suggesting a direct “hit” by TDP‐43 deposition.